# *O*-GlcNAcylation: the sweet side of epigenetics

**DOI:** 10.1186/s13072-023-00523-5

**Published:** 2023-12-14

**Authors:** Thomas Dupas, Benjamin Lauzier, Serge McGraw

**Affiliations:** 1 Centre Hospitalier Universitaire Sainte-Justine Research Center, Montréal, Canada; 2 Department of Obstetrics and Gynecology, Université de Montréal, 2900 Boulevard Edouard‑Montpetit, Montréal, QC H3T 1J4 Canada; 3 Nantes Université, CNRS, INSERM, L’institut du Thorax, 44000 Nantes, France

**Keywords:** *O-*GlcNAcylation, Epigenetics, Histone modification

## Abstract

Histones display a wide variety of post-translational modifications, including acetylation, methylation, and phosphorylation. These epigenetic modifications can influence chromatin structure and function without altering the DNA sequence. Histones can also undergo post-translational *O-*GlcNAcylation, a rather understudied modification that plays critical roles in almost all biological processes and is added and removed by *O-*linked *N-*acetylglucosamine transferase and *O-*GlcNAcase, respectively. This review provides a current overview of our knowledge of how *O*-GlcNAcylation impacts the histone code both directly and by regulating other chromatin modifying enzymes. This highlights the pivotal emerging role of *O-*GlcNAcylation as an essential epigenetic marker.

## Introduction

**Fig. 1:**
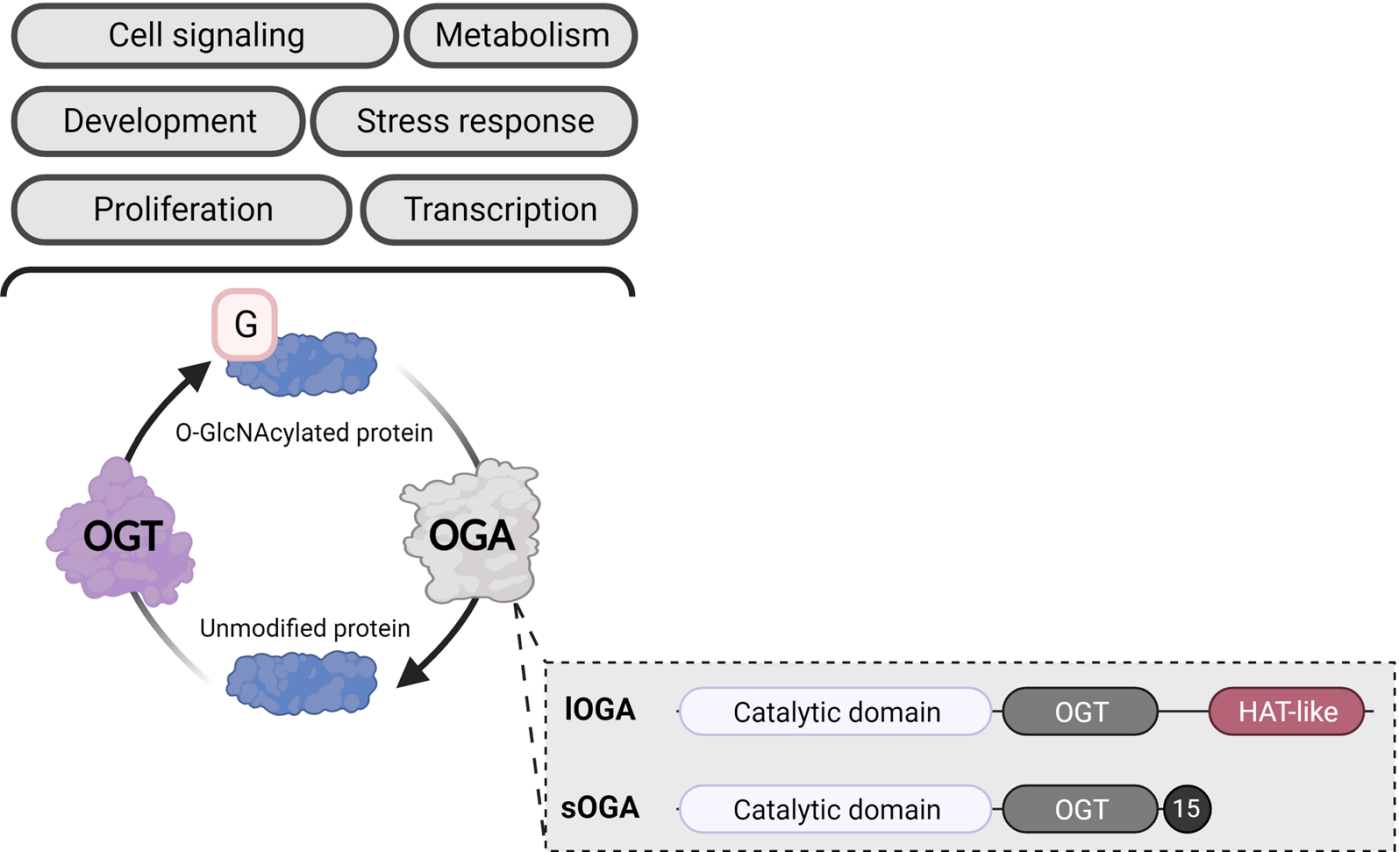
O-GlcNAcylation consists in the addition of a GlcNAc moiety on proteins which are involved in many if not all cellular processes. OGA exists in two isoforms: long OGA (lOGA) has a histone-like acetyltransferase domain (HAT-like), while short OGA (sOGA) does not. Both isoforms contain a catalytic domain and an OGT-binding domain (OGT). The C-terminal of sOGA contains a specific sequence of 15 amino acids (15). Created with BioRender.com

Gene expression is influenced by physiological (e.g*.*, cell differentiation, development and aging, external stressors) and pathological (e.g*.*, cancer, neurodegenerative diseases) factors [1]. Several cellular processes can also impact gene expression, including transcription, mRNA stability and transport, and translation [2]. Epigenetic modifications provide an important layer of regulation, altering gene expression without changing the DNA sequence [3]. The best-described epigenetic mechanism is the addition of biochemical marks directly to the DNA or the histone proteins that organize it. Cytosine methylation to form 5-methylcytosine is the most common chemical DNA base modification, although additional changes (e.g., 5-hydroxymethylcytosine, N6-methyladenine) have been recently discovered [4]. Covalent changes to histones, known as post-translational modifications (PTMs), include methylation (me), phosphorylation, acetylation (ac), ubiquitylation, SUMOylation, glycosylation, and ADP-ribosylation [5]. In 2010, Sakabe et al*.* added a new histone PTM: *O-*GlcNAcylation (*O-*GlcNAc) [6]—the ubiquitous, dynamic, and reversible addition of a sugar motif (β-D-*N-*acetylglucosamine) to serine and threonine residues. The *O-*GlcNAcylation cycle is controlled by a single pair of enzymes: *O-*linked *N-*acetyl-glucosaminyltransferase (OGT) adds the GlcNAc moiety to proteins, while *O-*linked *N-*acetyl β-D-glucosaminidase (OGA) removes it (Fig. 1) [7].

**Fig. 2:**
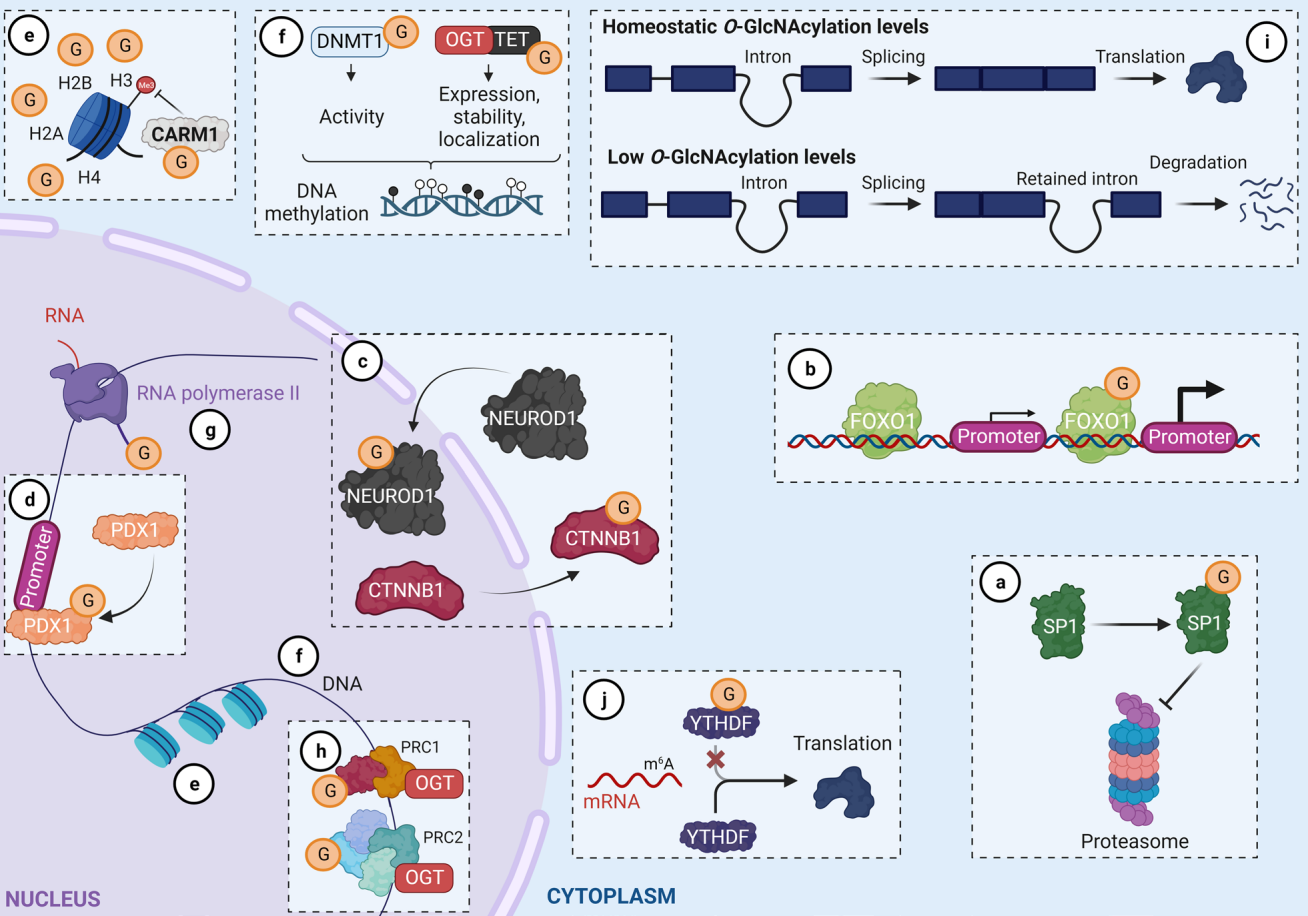
O-GlcNAcylation is involved in the regulation of gene expression through different mechanisms. O-GlcNAcylation regulates stability (**a**), transcriptional activity (**b**), localization (**c**) and DNA binding (**d**) of transcription factors; impacts directly or indirectly histone modification (**e**); modulates DNA methylation (**f**); regulates RNA polymerase II (**g**); controls PRC (**h**); contributes to the intro retention process (**i**) and determines translational regulation of N^6^-methyladenosine (m^6^A) modified-mRNAs (**j**). PRC1, PRC2: Polycomb repressive complex 1, 2; CARM1: coactivator-associated arginine methyltransferase 1; DNMT1: DNA methyltransferase 1; TET: ten-eleven translocation; SP1: transcription factor SP1; PDX1: Pancreas/duodenum homeobox protein 1; CTNNB1: catenin beta-1; NEUROD1: transcription factor NEUROD1; FOXO1: forkhead box protein O1; YTHDF: YTH m6A-RNA-binding proteins. Adapted from Brimble et al., Tan et al., and Dehennaut et al. Created with BioRender.com

**Fig. 3:**
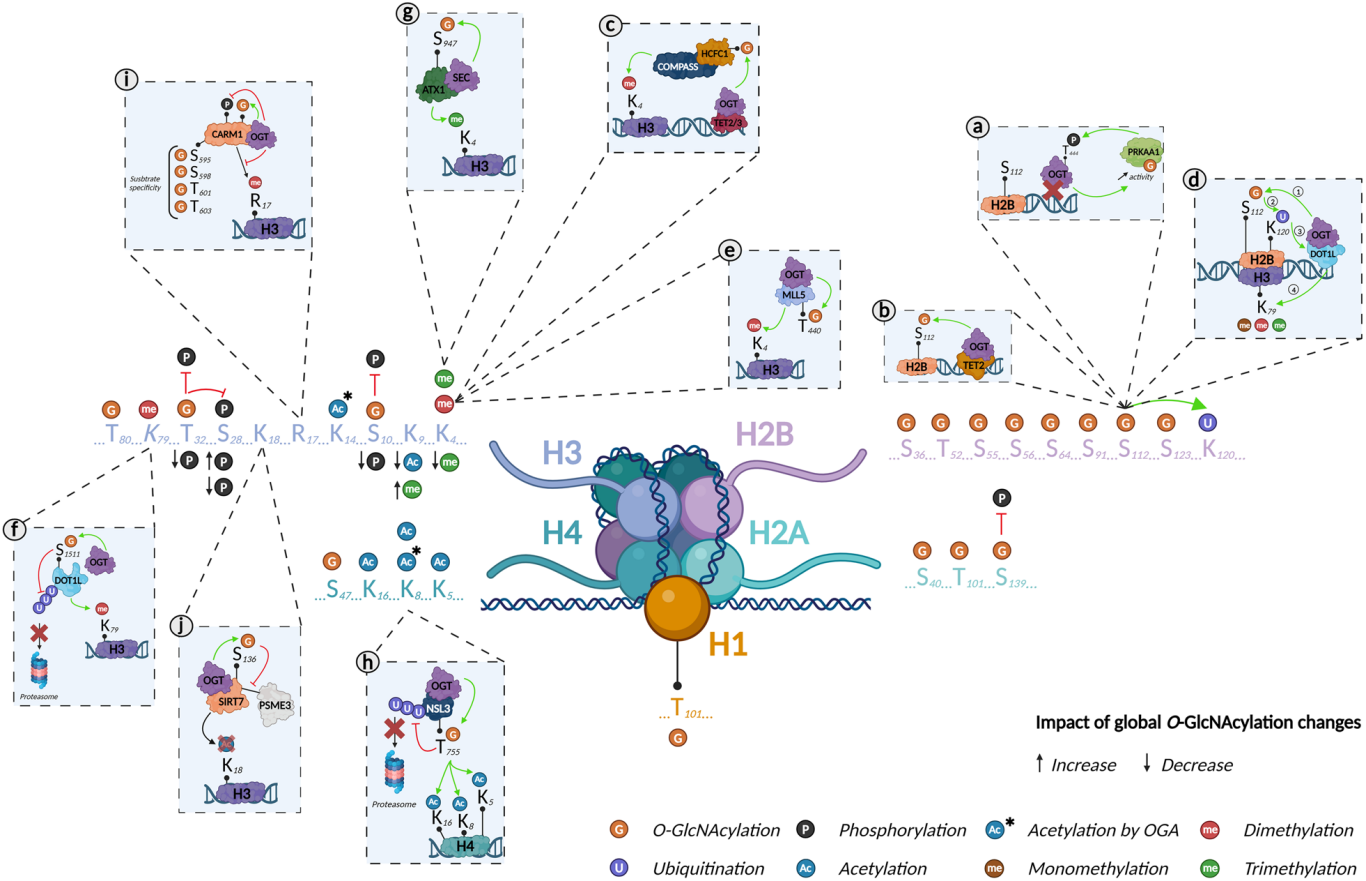
O-GlcNAcylation as an essential component of the histone code. Representation of all O-GlcNAcylated histone sites (**b**, **d**) and impact of O-GlcNAcylation on other histone marks via its effect on writers/erasers proteins (**a**, **c**, **d**, **e**, **f**, **g**, **h**, **i**, **j**). Created with BioRender.com

*O-*GlcNAcylation helps regulate gene expression by (1) changing the properties of transcription factors (localization, stability, DNA binding, and transcriptional activity; Fig. 2a–d); (2) directly or indirectly modifying histones (Fig. 2e); (3) impacting DNA methylation through modulation of DNA methyltransferase 1 (DNMT1) and ten–eleven translocation 1, 2 and 3 (TET1, 2 and 3) protein properties (activity for DNMT; stability and DNA binding for TET) (Fig. 2f); and (4) regulating RNA polymerase II transcription at the initiation and elongation stages (Fig. 2g) [8–12]. Moreover, OGT interacts with and regulates proteins in polycomb repressive complexes (PRCs) 1 and 2 [13], and a recent study reported that *O-*GlcNAcylation levels contribute to the intron retention process (Fig. 2h, i) [14]. Finally, as evidence of its broad impact on gene expression, *O*-GlcNAcylation dictates the translational regulation of mRNAs modified with *N*^*6*^-methyladenosine (m^6^A) through YTH domain-containing m6A-RNA-binding proteins (Fig. 2j) [15]. Recently developed approaches have enabled considerable progress in identifying *O-*GlcNAcylated proteins and in unraveling the role of *O-*GlcNAcylation in numerous biological processes [16]. To date, the set of *O-*GlcNAcylated proteins in humans, known as the *O-*GlcNAcylome, consists of 8000 proteins and continues to grow (The *O-*GlcNAc Database, v1.3) [17]. This review provides an updated look at its role as an epigenetic marker, focusing on histone modifications.

## Histone *O-*GlcNAcylation

In eukaryotic nuclei, the DNA is wrapped around a histone octamer (containing two copies each of histones H2A, H2B, H3, and H4), to form a nucleosome, and is locked by histone H1. The broad spectrum of histone PTMs constitutes the “histone code”, which not only modulates the recruitment of key enzymes involved in gene expression, but also impacts the condensation of chromatin. This results in distinct areas of euchromatin, which is only slightly condensed and transcriptionally active, and highly condensed and transcriptionally silent heterochromatin [19, 20].

### Initial evidence

Histone *O-*GlcNAcylation was first reported in 2010. Using several biochemical and mass spectrometry (MS) approaches, Sakabe et al*.* revealed that H2A, H2B, H3, and H4 were *O-*GlcNAcylated (at T101, S36, and S47 in H2A, H2B, and H4, respectively; the modified site on H3 was not identified) in HeLa cells [6]. They demonstrated that heat stress was associated with increased histone *O-*GlcNAcylation, concomitant with DNA condensation. This discovery created a novel field of research on *O-*GlcNAcylation*-*mediated stress responses and added a new layer of complexity to the histone code. The following year, Hahne et al. mapped additional *O-*GlcNAcylated sites on H2B (T52, S55, S56, and S64) using a bioinformatics analysis tool called Oscore, which detects and ranks tandem MS spectra by their probability of containing *O-*GlcNAc peptides (Fig. 3**, **Table 1) [21]. However, these *O-*GlcNAc sites have not yet been confirmed by other studies.

### Histone *O-*GlcNAcylation throughout evolution

*O-*GlcNAcylated histones have also been reported in plants indicating that the mechanism is conserved across diverse phyla. Schouppe et al*.* identified three new *O-*GlcNAcylated sites in cultured cells from *Nicotiana tabacum* cv. Xanthi, on H1 (T101), H2B (S65), and H3.3 (T80) [22]. However, *O-*GlcNAcylation sites can differ among species. Using MS, Hirosawa et al. mapped *O-*GlcNAcylation to S40 of H2A. This PTM occurred specifically in viviparous species, which expressed both H2A S40 and H2A A40 isoforms, while more phylogenetically distant species expressed only the A40 isoform [23]. This study demonstrated that epigenetic processes/machineries are not fully conserved between vertebrates, pinpointing the existence of species-dependent regulatory mechanisms and limiting the use of particular animal models, depending on the scientific hypothesis (*e.g*., zebrafish are commonly used as a model for epigenetic studies but lack the H2A S40 isoform; Fig. 3, Table 1) [24]

### Histone *O-*GlcNAcylation and DNA damage repair process

**Fig. 4:**
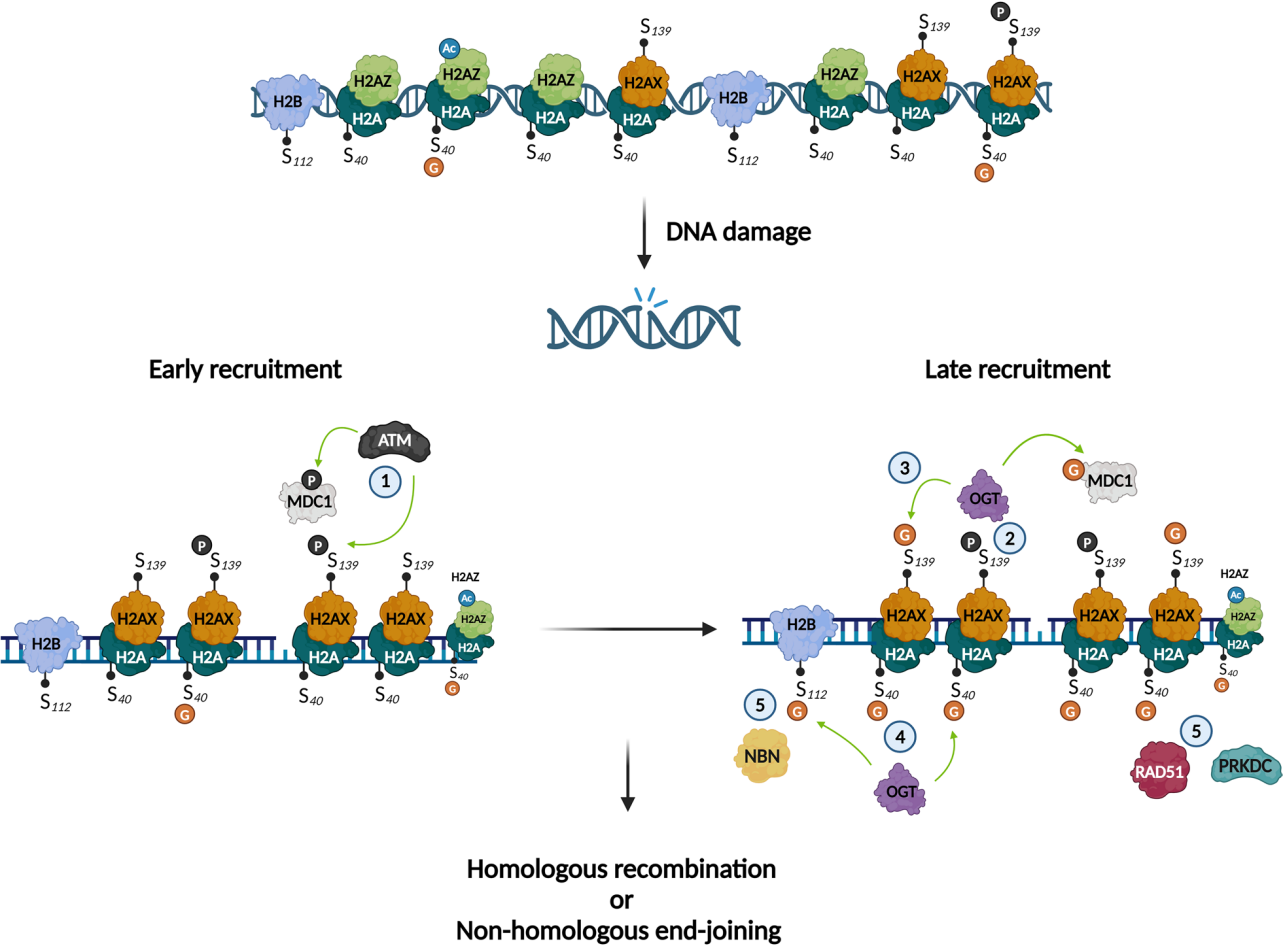
Histone O-GlcNAcylation as a key mechanism in DNA damage repair. ① Kinase ATM phosphorylates H2AX and MDC1; ② γH2AX recruits OGT and favors O-GlcNAcylation of H2AX on S139 and MDC1; ③ GlcNAc-H2AX restrains γH2AX expansion around the DNA-damaged site; ④ OGT O-GlcNAcylates H2A on S40 and H2B on S112; ⑤ GlcNAc-H2A favors accumulation of PRKDC and RAD51, while GlcNAc-H2B favors the accumulation of NBN. Adapted from Chen and Yu, Hayakawa et al. and Wang et al. [25, 26, 30]. Created with BioRender.com

DNA damage, caused by endogenous (e.g., reactive oxygen species, water) or exogenous (e.g*.*, UV radiation or ionizing radiation) sources, can impact health. Accordingly, cells have developed several response mechanisms to maintain the DNA’s integrity. Hayakawa et al. provided evidence that *O-*GlcNAcylation of H2A S40 is involved in DNA damage repair, by interacting with phosphorylated H2AX (γH2AX) and acetylated H2AZ (AcH2AZ) and recruiting the key DNA repair enzymes protein kinase, DNA-activated catalytic subunit (PRKDC), and RAD51 recombinase (RAD51) [25]. As the H2A S40 isoform is species dependent, this study reinforces the existence of distinct DNA repair mechanisms between species. S139 on H2AX can also undergo *O-*GlcNAcylation [26]. Interestingly, Chen et al*.* showed that OGT was recruited by S139-phosphorylated H2AX, promoting *O-*GlcNAcylation of H2AX close to sites of damage, thus delimiting the expansion territory of γH2AX. They also determined that the mediator of DNA damage checkpoint 1 (MDC1) was *O-*GlcNAcylated. As phosphorylation of both MDC1 and H2AX prolongs G2/M arrest and can eventually cause apoptosis, the authors suggested that *O-*GlcNAcylation of MDC1 and H2AX helps cells recover from DNA damage. Finally, Wang et al*.* demonstrated that DNA damage induction led to local increases in the *O-*GlcNAcylation of H2B S112. They suggested a mechanism in which H2B S112 *O-*GlcNAcylation regulates DNA damage repair via interaction with nibrin (NBN), which is involved in DNA double-strand break repair and DNA damage-induced checkpoint activation. They also showed that H2B S112 *O-*GlcNAcylation promoted NBN accumulation at damaged DNA sites, but was not involved in the interaction with γH2AX [30]. Collectively, these results indicate several mechanisms that could explain the beneficial effects of *O-*GlcNAcylation in DNA repair (Fig. 4**, **Table 1).

**Table 1:**
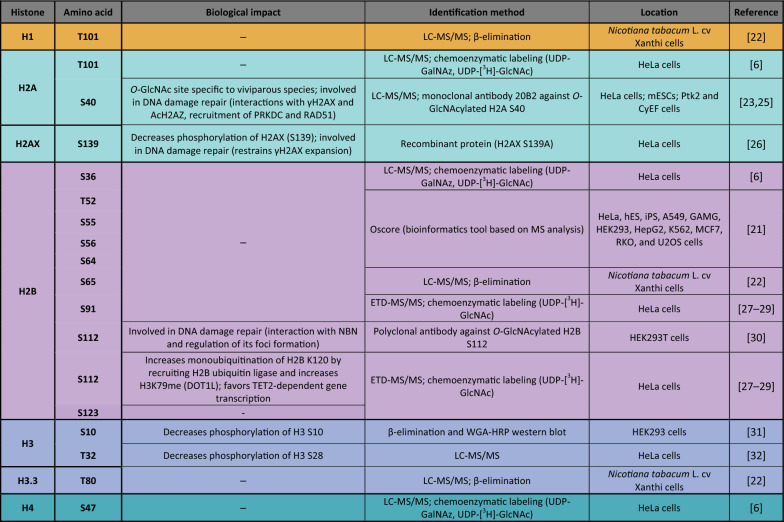
Identified *O-*GlcNAcylated histone residues

*LC–MS/MS* liquid chromatography–tandem mass spectrometry, *UDP-GalNAz* uridine diphosphate N-azidoacetylgalactosamine, *WGA–HRP* wheat germ agglutinin conjugated to horseradish peroxidase

### Impact of histone *O-*GlcNAcylation on gene expression

Like other PTMs in the histone code, *O-*GlcNAcylation serves to regulate gene expression. Using electron transfer dissociation (ETD)-MS/MS, Fujijki and colleagues identified several *O-*GlcNAcylation sites on H2B (S91, S112, and S123) and H2A (T101). Surprisingly, they were unable to detect the previously reported sites on H2B (S36) and H4 (S47). They showed that H2B (S112) *O-*GlcNAcylation facilitates the recruitment of the H2B ubiquitin ligase, which led to H2B monoubiquitination on lysine 120 (K120). More interestingly, they found that *O-*GlcNAcylation of H2B S112 (H2BS112G) can co-occur with the active H3K4me2 mark, suggesting that H2BS112G is involved in transcriptional activation [27]. Consistent with this hypothesis, Xu et al*.* showed that despite not impacting OGT’s activity, protein kinase AMP-activated catalytic subunit alpha 1 (PRKAA1)-dependent phosphorylation of T444 inhibits OGT’s association with the chromatin and, therefore, H2BS112G deposition and gene expression. They also revealed the existence of a positive feedback loop, in which *O-*GlcNAcylation of PRKAA1 increases its activity (Fig. 3a) [33].

Ten–eleven translocation (TET) proteins are key enzymes implicated in removing DNA methylation marks that impact gene expression. Chen et al*.* demonstrated that *O-*GlcNAcylation of H2B S112 occurs after TET2 recruits OGT, and thus that histone *O-*GlcNAcylation participates in TET2-dependent gene transcription (Fig. 3b) [28]. This result was supported by Deplus et al., who reported that TET2/3-mediated OGT recruitment promoted *O-*GlcNAcylation of host cell factor C1 (HCFC1), an important protein for the formation of COMPASS, a methyltransferase that deposits the active epigenetic mark H3K4me3 (Fig. 3c) [34]. Recently, Xu et al. reported that DOT1 like histone lysine methyltransferase (DOT1L), which deposits mono-, di-, and tri-methylated marks on H3K79, acts as a scaffold protein that enables OGT’s recruitment to the chromatin. They suggested that OGT recruitment via DOT1L favors H2BS112G deposition, which facilitates the ubiquitination of H2BK120, a modification that stimulates DOT1L activity to increase H3K79me (Fig. 3d) [29]. Overall, these results highlighted that *O-*GlcNAcylation plays a pivotal role in gene transcription through histone modification through complex, multi-layered mechanisms (Table 1).

In addition, *O-*GlcNAcylation actively influences gene accessibility by modulating the open/closed state of chromatin and the recruitment of key enzymes. Lercher et al*.* demonstrated that *O-*GlcNAcylation of H2A T101 decreased nucleosome stability, favoring an open state and thus promoting the recruitment of proteins involved in nucleosome remodeling (e.g., mutS homologs 2 and 6) [35]. Taken together, these studies illustrate that OGT and *O-*GlcNAcylation are full-fledged players in histone modification.

### Interplay between histone *O-*GlcNAcylation and phosphorylation

As they target the same amino acids (serine and threonine), phosphorylation and *O-*GlcNAcylation are closely linked and can compete against each other [36]. Moreover, *O-*GlcNAcylation can positively or negatively regulate the phosphorylation of nearby residues, and OGT/OGA can interact with kinases/phosphatases, creating multi-enzyme complexes that can phosphorylate/*O-*GlcNAcylate proteins [37–39]. Lowndes’ group revealed that H3 was *O-*GlcNAcylated, which partially supressed its phosphorylation. They also highlighted that increasing *O-*GlcNAc levels via glucosamine was associated with decreases in both H3K9ac and H3K4me3—both active marks—and increases in H3 S28 phosphorylation and H3K9me3, which are active and repressive, respectively [31]. In 2012, Fong et al*.* determined that H3 T32 was *O-*GlcNAcylated. As aurora B, the kinase that phosphorylates H3 S10 and S28, is physically associated with OGT/OGA, they evaluated the impact of *O-*GlcNAc levels on these phosphosites. Mitotic cells that overexpressed OGT or were treated with OGA inhibitors (PUGNAc or thiamet G) displayed reduced H3 S10, S28, and T32 phosphorylation (Fig. 3, Table 1) [32]. Interestingly, no changes in H3S28 phosphorylation were observed in PUGNAc-treated asynchronous cells. This inconsistency with the previous study may stem from the treatments used to increase *O-*GlcNAcylation levels. Compared with PUGNAc or thiamet G, glucosamine is less specific and has been associated with off-target effects that interfere with proteoglycan and ATP production [40]. Regardless, considering that H3 S10 and S28 phosphosites are associated with chromatin condensation during mitosis, these two studies provide general evidence that *O-*GlcNAcylation regulates the cell cycle by competing with phosphorylation of H3 at different sites.

### *O-*GlcNAcase as a histone acetyltransferase?

The major role of *O-*GlcNAcylation in histone modification is reinforced by OGA’s C-terminal HAT activity [41]. Toleman et al*.* demonstrated that mammalian OGA can acetylate all four core histones in synthetic nucleosomes in vitro. They also identified an *O*-GlcNAcylated site for H4 (K8) and H3 (K14) (Fig. 3). Interestingly, bacterial OGA lacked acetyltransferase activity, except when the enzyme was incubated with mammalian proteins, suggesting the existence of mammalian-specific regulation. Two years later and through a series of biochemical strategies, the researchers involved in the previous study discovered that the OGA contains a zinc finger-like domain that ensures histone binding [42]. However, Butkinaree et al*.* extensively demonstrated that OGA lacked histone acetyltransferase activity [43]. Consistent with this, Rao et al. demonstrated that human OGA lacks the key amino acids for both histone acetyltransferase and acetyl-CoA binding [44]. Considering these controversial results, the C-terminal extremity is now qualified as a histone acetyltransferase (HAT)-like domain. Interestingly, this domain is only present as the long OGA isoform (in the short isoform, the HAT-like domain is deleted and replaced by a specific 15-residue sequence), suggesting a specific role; however, this role remains unknown (Fig. 1) [45].

## *O-*GlcNAcylation indirectly affects histones via chromatin modifying enzymes

Like many proteins, chromatin modifying enzymes are regulated in part by PTMs such as *O-*GlcNAcylation, impacting their expression, activity, interactomes, and stability (Table 2). Several proteins that add (“writers”) and remove (“erasers”) various histone or DNA marks interact with OGT and/or are *O*-GlcNAcylated, highlighting the importance of *O-*GlcNAcylation [18, 29, 46–49].

**Table 2:**
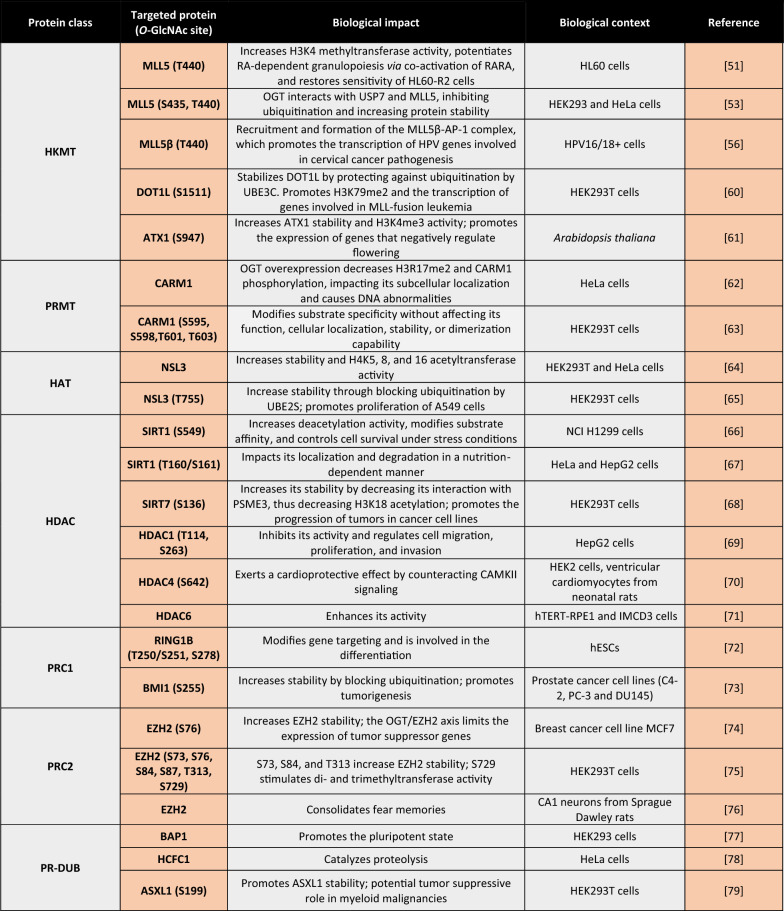
Summary of O-GlcNAcylated histone writers/erasers

### *O-*GlcNAcylation of histone writers

The protein mixed leukemia lineage 5 (MLL5) is a histone lysine methyltransferase (HKMT) involved in regulating cell cycle progression, spermatogenesis, hematopoiesis, and the maintenance of genomic stability [50]. Fujiki et al. demonstrated that OGT binds and *O-*GlcNAcylates MLL5 on T440. This increased MLL5’s H3K4 methyltransferase activity to potentiate retinoic acid (RA)-induced granulopoiesis (Fig. 3e). Interestingly, HL60-R2 cells, which are resistant to granulopoiesis, displayed high *O-*GlcNAcase activity compared with granulopoiesis-sensitive HL60 cells. In addition, the inhibition of OGA with PUGNAc restored responses to RA and thus the methylation of H3K4, suggesting a direct role of *O-*GlcNAcylation in granulopoiesis [51]. More recently, Ding et al*.* showed that MLL5 stability was cooperatively controlled by OGT and ubiquitin-specific protease 7 (USP7). They showed that the three proteins interacted, limiting the ubiquitination and thus the degradation of MLL5. Although the authors identified two *O-*GlcNAcylated sites on MLL5 (S435 and T440), they did not determine their roles in protein stability. Moreover, as USP7 can be phosphorylated [52], it would be relevant to evaluate if it is also *O-*GlcNAcylated and how that would impact its functions [53]. Finally, the authors demonstrated that the increased MLL5 levels were similar to increase in both OGT and USP7 observed in cervical adenocarcinomas [54, 55]. Consistently, Nin et al*.* demonstrated that *O-*GlcNAcylation of MLL5 on T440 favors the recruitment of MLL5β to the MLL5β-AP-1 complex, which allows the transcription of human papillomavirus E6/E7 oncogenes implicated in the pathogenesis of cervical cancer [56]. In addition to being implicated in cervical cancer, chromosome translocations involving *MLL* can cause MLL-fusion leukemia, in which an MLL N terminus is fused to another protein. AF9, AF10, and ENL, which all interact with the H3K79 methyltransferase DOT1L, are the most common MLL fusion partners [57–59]. In 2021, Song et al. demonstrated that DOT1L was *O-*GlcNAcylated on S1511, which promotes its stability by protecting it from UBE3C-mediated ubiquitination (Fig. 3f). The authors also showed that OGT knockdown was associated with a decrease in H3K79me2 levels and the enrichment of HOXA9/MEIS1 mRNA (genes involved in the initiation and progression of the disease) and H3K79me2/DOT1L on the HOXA9/MEIS1 promoter, illustrating the role of DOT1L *O-*GlcNAcylation in MLL fusion leukemia pathogenesis [60]. Taken together, these studies highlight the important role of *O-*GlcNAcylation in cancer pathogenesis—regulating ubiquitin*-*mediated degradation and gene expression.

*O-*GlcNAcylation also controls HKMTs in plants. The *Arabidopsis* homolog of trithorax (ATX1) is a H3K4 methyltransferase. Xing et al*.* demonstrated that secret agent (SEC), the OGT in *Arabidopsis*, regulated both the stability and activity of ATX1 through *O-*GlcNAcylation of S947. They also demonstrated that *O-*GlcNAcylation of ATX1 promotes H3K4me3 deposition on *FLOWERING LOCUS C,* which encodes key negative regulators of flowering (Fig. 3g) [61]. Considering *O-*GlcNAcylation’s reported roles in protein degradation, it would be interesting to identify the mechanism by which it regulates ATX1.

In addition to HKMTs, *O-*GlcNAcylation can also modify the properties of other histone writers. The histone lysine acetyltransferase 8 (KAT8) contains male-specific lethal and nonspecific lethal (NSL) complexes. Interestingly, OGT1 is a component of the NSL complex [80]. In 2017, Wu et al*.* demonstrated that OGT1 interacted with and *O-*GlcNAcylated the NSL complex subunit NSL3, which was associated with increased stability and activity, thereby facilitating H4 acetylation on K5, K8, and K16 [64]. More recently, the same group used MS and several biochemical methods to identify *O-*GlcNAcylation of T755 of NSL3, which increased NSL3 stability by blocking UBE2S-dependent ubiquitination. Even more importantly, *O-*GlcNAcylated T755 was required to maintain the integrity and holoenzyme activity of the NSL complex. In type II epithelium-like lung carcinoma (A549) cells, NSL3 *O-*GlcNAcylation promoted proliferation, leading them to conclude that *O-*GlcNAcylation acts as a link between oncogenic signals and the epigenetic changes that occur in cancer (Fig. 3h) [65].

Coactivator-associated arginine methyltransferase 1 (CARM1), as also known as protein arginine *N-*methyltransferase 4 (PRMT4), is an enzyme that asymmetrically dimethylates proteins on arginine residues. It is both a substrate and interactor of OGT [81]. In 2010, Sakabe and Hart demonstrated that OGT overexpression not only decreased H3R17me2 (a CARM1-specific target) and that CARM1 phosphorylation impacted its subcellular localization, resulting in DNA abnormalities (e.g., errors in chromosomal separation, chromosomal bridges). Interestingly, increasing *O-*GlcNAcylation levels via thiamet G or *N*-acetyl-glucosamine-thiazoline did not impact H3R17me2 levels or CARM1 phosphorylation, suggesting the direct involvement of OGT [62]. Later, Charoensuksai et al*.* identified four *O-*GlcNAcylated sites on CARM1: S595, S598, T601, and T603. They showed that these modifications impacted CARM1’s substrate specificity without affecting its function, cellular localization, stability, or dimerization (Fig. 3i) [63]. As dysregulated CARM1 expression and/or activity of CARM1 has been described in various pathologies [82], it would be interesting to determine the impact of *O-*GlcNAcylation and OGT on CARM1.

### *O-*GlcNAcylation of histone erasers

Histone deacetylases (HDACs) catalyze the removal of acetyl groups from both histones and non*-*histone proteins. Humans express 18 HDACs (HDAC1–11, SIRT1–7) [83]. To date, three (HDAC1, 4, and 6) have been identified as *O-*GlcNAcylated. Zhu et al*.* showed that OGT interacted with and *O-*GlcNAcylated HDAC1 on T114 and S263, which suppresses HDAC1 enzymatic activity. They also showed that HDAC1 *O-*GlcNAcylation regulates the migration, proliferation, and invasion of HepG2 cells, thus identifying a new potential therapeutic strategy for hepatocellular carcinoma [69]. More recently, HDAC4 was reported to be *O-*GlcNAcylated on S642. In the diabetes mellitus mouse model, this *O-*GlcNAcylation event counteracted pathological CAMKII signaling and thus was deemed cardioprotective [70]. Considering these important effects, it would be relevant to evaluate if and how HDAC4 *O-*GlcNAcylation impacts the histone acetylation landscape. HDAC6 plays a pivotal role in cilia assembly and is regulated by phosphorylation [84]. Considering the widely reported cross talk between phosphorylation and *O-*GlcNAcylation [85], Tian and Qin examined if HDAC6 was also regulated by *O-*GlcNAcylation. They discovered that OGT interacted with and *O-*GlcNAcylated HDAC6 in hTERT-RPE1 cells, resulting in ciliary shortening, and demonstrated that treatment with the OGT inhibitors thiamet G or GlcNAcstatin enhanced HDAC6’s deacetylase activity [71]. To date, the effects of these *O-*GlcNAcylated HDACs on histone acetylation have not been examined.

Sirtuins (SIRTs) are nicotine adenine dinucleotide( +)-dependent HDACs that regulate a wide variety of biological processes, such as metabolism, oxidative stress, apoptosis, and inflammation [86]. SIRT1 is a critical stress sensor that regulates both histones and non*-*histone proteins (e.g., p53, NFκB, eIF2α). Interestingly, *O-*GlcNAcylation is also pivotal in the stress response [87]. Consistent with this overlap, Han et al. demonstrated that SIRT1 interacted directly with OGT. Using chemoenzymatic and metabolic labeling coupled with MS, they showed that SIRT1 was *O-*GlcNAcylated on S549, which enhanced its deacetylase activity (evaluated on histone H3 and cellular tumor antigen p53 (p53)) and its substrate affinity (evaluated on p53). They found that under stress, SIRT1 *O-*GlcNAcylation allowed some targets to be deacetylated, including p53 and FOXO3, which regulate the decisions governing cell death and survival [66]. Han et al*.* found that SIRT1 *O-*GlcNAcylation on S549 did not affect its subcellular localization, and a recent study reported complementary results. In fact, Chattopadhyay et al. revealed that the nutrient-dependent SIRT1 *O-*GlcNAcylation of T160/S161 exerts spatiotemporal control by promoting its localization to the cytosol, where it undergoes ubiquitin*-*mediated degradation [67]. Considering these results, evaluating if SIRT1 *O-*GlcNAcylation impacts the acetylation status of histones would be worth exploring. SIRT7, which catalyzes the selective deacetylation of H3K18, was recently identified as *O-*GlcNAcylated by OGT at S136, which stabilizes SIRT7 by decreasing its interaction with proteasome activator subunit 3 (PSME3), a core molecule of a new ubiquitin*-*independent pathway [88]. By reducing H3K18Ac, SIRT7 *O-*GlcNAcylation has been associated with repressing tumor suppressor genes to promoting tumor progression in nude mice (Fig. 3j) [68].

## Focus on *O*-GlcNAcylation of polycomb repressive complexes

The polycomb group (PcG) proteins form three complexes known as PRC1, PRC2, and PR DeUBiquitinase (PR-DUB). PRCs play a pivotal role in development by repressing homeotic genes. Mutations in the subunits of PRCs are associated with human neurodevelopmental disorders and cancer [89, 90]. In mice, deleting PcG genes causes embryonic lethality [91]. PRC1 monoubiquitinates K119 of histone H2A, which can be removed by PR-DUB. PRC2 is a HKMT that mono*-*, di-, and trimethylates H3K27 to form H3K27me1, me2, and me3, respectively. *O-*GlcNAcylation plays an important role in gene regulation in *Drosophila*. As proof, OGT is encoded by the PcG gene super sex combs (*sxc*) [92], and loss of OGA leads to global perturbation of the epigenetic machinery [93]. Although PRC1, PRC2, and PR-DUB exist in *Drosophila*, no major features have been identified recently [13].

**Fig. 5:**
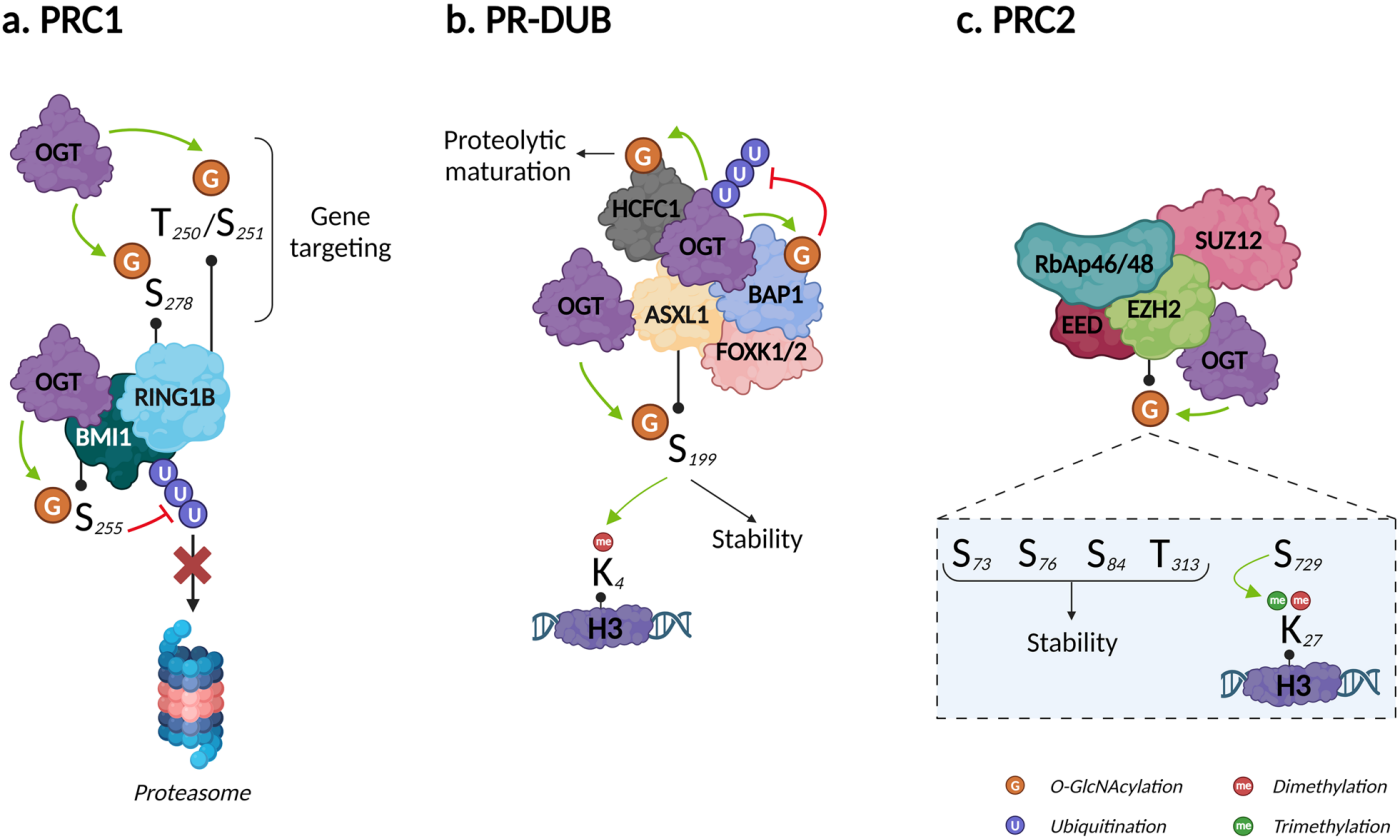
Impact of O-GlcNAcylation on Polycomb repressive complexes. **a** PRC1. O-GlcNAcylation on RING1B at T250/S251 and S278 controls PRC1 gene targeting, while O-GlcNAcylation on BMI1 at S255 increases its stability by limiting ubiquitin dependent proteasomal degradation; **b** PR-DUB. HCFC1 O-GlcNAcylation and OGT are indispensable for HCFC1 proteolytic maturation; BAP1 stabilizes OGT through limiting ubiquitin dependent proteasomal degradation; BAP1 is O-GlcNAcylated with no evidence of its impact. ASXL1 O-GlcNAcylation at S199 increases its stability; **c** PRC2. O-GlcNAcylation on EZH2 at S73, S76, S87 and T313 increases its stability while O-GlcNAcylation at S729 promotes its di- and trimethyltransferase activity. Created with BioRender.com

The PRC1 E3 ubiquitin protein ligase can be either RING1A or RING1B, which is associated with one of six PcG ring finger proteins (PCGF1–6). In contrast to RING1A, RING1B can be *O-*GlcNAcylated. Using MS coupled to beta-elimination and Michael addition with dithiothreitol, Maury et al*.* identified S278 as an *O-*GlcNAcylation site. They also found that T250 and S251 were important for *O-*GlcNAcylation, but could not discriminate which residue harbored the GlcNAc moiety. Using chromatin immunoprecipitation coupled with sequencing (ChIP-Seq), the authors revealed that RING1B *O-*GlcNAcylation impacted its target genes: *O-*GlcNAcylated RING1B was bound to genes involved in neuronal differentiation, while unmodified RING1B was bound to genes related to cell cycle and metabolism. Accordingly, unmodified RING1B decreased throughout human embryonic stem cell (hESC) differentiation [72], reinforcing the described role of *O-*GlcNAcylation in differentiation [94]. RING1B and BMI1 (also known as PGCF4) are all closely related to prostate cancer [95]. BMI1 phosphorylation protects it from proteasomal degradation [96]. In 2017, Li et al*.* demonstrated that OGT interacts with BMI1 and *O-*GlcNAcylates it on S255. Like phosphorylation, *O-*GlcNAcylation increased BMI1’s stability by inhibiting its polyubiquitination and proteasomal degradation. They also illustrated the negative role of BMI1 *O-*GlcNAcylation in prostate cancer tumorigenesis, as it inhibits p53, PTEN, and CDKN1A/CDKN2A signaling, thus favorizing apoptosis, invasion and proliferation, respectively (Fig. 5a) [73]. Although both RING1B and BMI1 are *O-*GlcNAcylated, there is no evidence that this impacts ubiquitin ligase activity; however, BMI1 was recently shown to regulate PRC1 ubiquitin ligase activity, which could be modulated by *O-*GlcNAcylation [97].

PR-DUB is a complex composed of BAP1, HCFC1, FOXK1/2, either ASXL1, 2, or 3, and interestingly, OGT [98]. In splenocytes, PR-DUB’s catalytic subunit BAP1 regulates HCFC1 and OGT and thus *O-*GlcNAcylation levels through its deubiquitinase activity [99]. Since OGT and *O-*GlcNAcylation are indispensable for the proteolytic maturation of HCFC1 [78], BAP1 (i) directly regulates HCFC1 expression and (ii) favors its maturation by stabilizing OGT. BAP1 *O-*GlcNAcylation was demonstrated only recently, with no evidence of impacts on its expression/stability or its deubiquitinase activity [77]. The close relationships between OGT, HCFC1, and BAP1 are exemplified by their roles modulating peroxisome proliferator-activated receptor gamma coactivator 1-alpha (PRGC1), a master regulator of gluconeogenesis. Ruan et al*.* demonstrated that HCFC1 recruits OGT to PRGC1 and that *O-*GlcNAcylation facilitates the binding, thus stabilizing PRGC1 and promoting gluconeogenesis [100]. ASXL1 is a component of PR-DUB, and also regulates H3K4me3 [101]. Recently, Inoue et al*.* identified ASXL1 as an OGT substrate that displayed increased stability after *O-*GlcNAcylation of S199 [79]. Disrupting the ASXL1–OGT complex reduced H3K4 methylation, indicating a pivotal tumor suppressive role for this signaling axis in myeloid malignancies (Fig. 5b).

PRC2’s catalytic activity is provided by three subunits: enhancer of zeste homolog 2 (EZH2), embryonic ectoderm development (EED), and suppressor of zeste 12 (SUZ12). The first evidence of the impact of EZH2 *O-*GlcNAcylation in humans was provided by Chu et al*.* in 2014. By treating two different cell lines with small interfering RNA, they demonstrated that OGT knockdown was associated with a decrease in H3K27me3 only. They showed that OGT (i) interacted with the EZH2/PRC2 complex, (ii) was essential for EZH2—and therefore, PRC2—stability, and (iii) *O-*GlcNAcylated EZH2 on S76. Finally, the authors demonstrated that the OGT/EZH2 axis downregulated tumor suppressor genes in breast cancer cells, thus identifying a new therapeutic target [74]. In 2018, using MS coupled to Click-iT^®^ *O*-GlcNAc enzymatic labeling system, Wong’s group identified five new *O-*GlcNAcylated sites on EZH2: S73, S84, S87, T313, and S729. After excluding S87, since *O*-GlcNAcylation at this site was very low, they showed that the N*-*terminal *O-*GlcNAcylated sites (S73, S84, and T313) increased the stability of EZH2 by limiting its ubiquitination, while the C-terminal *O-*GlcNAcylated site (S729) stimulates its di- and trimethyltransferase activity. None of the sites altered the affinity of EZH2 for other PRC2 components [75]. EZH2 has been shown to be involved in cancer [102]. In addition, Butler et al. recently demonstrated that OGT exerted control on histone regulation via EZH2-dependent H3K27me3 during the consolidation of fear memories [76]. To date, EZH2 is the only component of PRC2 identified as *O-*GlcNAcylated; therefore, it would be relevant to study whether *O-*GlcNAcylation also impacts SUZ12, EED, and RbAp46/48 (Fig. 5c).

## Perspectives and future directions

While many studies over the past decade have established that H2A, H2B, H3, and H4 are *O-*GlcNAcylated, evidence for H1 *O-*GlcNAcylation remains limited. In 2011, an *in silico* study proposed *O-*GlcNAcylation of H1’s serine and threonine residues; however, this study relied on YinOYang 1.2 predictive tools, which remain controversial [103, 104]. As H1 plays an important role in chromatin organization and its phosphorylation can destabilize its bond with the DNA [105], further exploration of the potential impacts of H1 *O-*GlcNAcylation in this context is warranted.

The influence of *O-*GlcNAcylation goes beyond histone modifying enzymes. The different TET isoforms (TET1, 2, and 3), which catalyze the oxidation of 5-methylcytosine to remove DNA methylation [106], also interact with OGT [34, 107]. These interactions and/or the *O-*GlcNAcylation of TET proteins affects their stability, phosphorylation, and DNA binding, and thus ability to remove DNA methylation [11, 12, 34, 107, 108]. While TET *O-*GlcNAcylation is well documented, the first evidence of *O-*GlcNAcylation of a DNA writer, DNA methyltransferase (DNMT), only emerged in 2020 [109]. Interestingly, OGT is enriched at the promoter of *DNMT3B*, which encodes one of two enzymes regulating de novo methylation, suggesting that its expression might be controlled [46]. Moreover, *O-*GlcNAcylation of DNMT1, the enzyme that ensures the transmission of DNA methylation patterns during replication, was recently shown to reduce its methyltransferase activity [10]. Given the central role of DNMTs in physiological (e.g., stem cell fate, cardiac metabolism, and contractility) and pathological (e.g., cancers, Tatton–Brown syndrome) conditions, as well as in developmental (e.g., DNA methylation reprogramming in early embryos, differentiation) processes unraveling how *O-*GlcNAcylation regulates these proteins could lead to promising research avenues [110–113]. From a research standpoint, this interplay between environmental factors (e.g., prenatal alcohol exposure, toxicants) and *O*-GlcNAcylation offers a promising avenue for exploring how external stimuli modulate gene expression and cellular responses via epigenetic regulation (e.g., DNA methylation, histone modifications) potentially unlocking new insights into the mechanisms of cellular adaptation and homeostasis during early development [114–116].

Developing tools and approaches to enhance our understanding of *O-*GlcNAcylation as an epigenetic mark is a major challenge in the field. Currently, only two antibodies against *O-*GlcNAcylated histones are available (for H2BS112 and H2AS40) [23, 30]. Although they are less expensive and labor intensive than specific antibodies, pan-*O*-GlcNAcylation antibodies have different selectivity and poor specificity [16]. Thus, expanding the antibody collection to detect all possible *O-*GlcNAcylated histone residues would allow better mapping of the modification using ChIP-Seq- or MS-based approaches. Current strategies to understand the role of *O-*GlcNAcylation include tissue-specific knockouts, RNA interference, and OGT and OGA inhibitors [16]. Considering the implications of *O-*GlcNAcylation in pathophysiological situations (e.g., developmental defects, sepsis), pairing these strategies with advanced epigenetic methods could clarify how *O-*GlcNAcylation interacts with other histone and DNA marks in various contexts [1, 117, 118]. Finally, identifying new *O-*GlcNAcylated sites on histones and epigenetic enzymes using MS, then preventing their modification via mutation, will enhance our understanding of the broad implications of *O-*GlcNAcylation [16].

## Conclusion

In this review, we have highlighted the important roles of *O-*GlcNAcylation on core histones and its cross talk with the other nucleosomal PTMs. It is very likely that known *O-*GlcNAcylated proteins represent only a fraction of the broader role it plays in epigenetics. By developing more refined methods, we will identify more proteins influenced by this modification. Notably, some studies have taken a more critical look at histone *O-*GlcNAcylation. For instance, Gambetta et al*.* emphasized the need to treat previous results on the role of *O-*GlcNAcylation in epigenetics with caution [119]. Moreover, certain recent findings raise questions of abundance and occurrence of *O*-GlcNAcylation on histones as well as presence of other factors for efficient *O*-GlcNAcylation [120, 121]. There is still much to uncover to understand the intricacies of *O-*GlcNAcylation’s roles in epigenetic regulation and determine how these roles can be targeted to improve human health, underscoring the importance of continued research in this area.

## Data Availability

Not applicable.

## Abbreviations

ATX1: Arabidopsis homolog of trithorax
CARM1: Coactivator-associated arginine methyltransferase 1
ChiP-Seq: Chromatin immunoprecipitation coupled with sequencing
CTNNB1: Catenin beta-1
DNMT1: DNA methyltransferase 1
DOT1L: Histone lysine methyltransferase
EED: Embryonic ectoderm development
EZH2: Enhancer of zeste homolog
FOXO1: Forkhead box protein O1.
HAT: Histone acetyltransferase domain
HCFC1: Host cell factor C1
HDAC: Histone deacetylase
HKMT: Lysine methyltransferase
KAT8: Histone lysine acetyltransferase 8
LC–MS/MS: Liquid chromatography–tandem mass spectrometry
lOGA: Long OGA
MDC1: Mediator of DNA damage checkpoint 1
MLL5: Mixed leukemia lineage 5
MS: Mass spectrometry
NEUROD1: Transcription factor NEUROD1
NSL: Nonspecific lethal
O-GlcNAc: O-GlcNAcylation
OGA: O-linked N-acetyl β-D-glucosaminidase
OGT: O-linked N-acetyl-glucosaminyltransferase
PDX1: Pancreas/duodenum homeobox protein 1
PRCs: Polycomb repressive complexes
PRGC1: Peroxisome proliferator-activated receptor gamma coactivator 1-alpha
PRKAA1: Protein kinase AMP-activated catalytic subunit alpha 1
PRKDC: DNA-activated, catalytic subunit
PRMT4: Protein arginine *N-*methyltransferase 4
PSME3: Proteasome activator subunit 3
PTM: Post-translational modification
sOGA: Short OGA
SP1: Transcription factor SP1
SUZ12: Suppressor of zeste 12
TET: Ten-eleven translocation
UDP-GalNAz: Uridine diphosphate N-azidoacetylgalactosamine
USP7: Ubiquitin-specific protease 7
WGA-HRP: Wheat germ agglutinin conjugated to horseradish peroxidase
YTHDF: YTH m6A-RNA-binding proteins
